# Multiplex PCR System for Rapid Detection of Pathogens in Patients with Presumed Sepsis – A Systemic Review and Meta-Analysis

**DOI:** 10.1371/journal.pone.0062323

**Published:** 2013-05-29

**Authors:** Shy-Shin Chang, Wen-Han Hsieh, Ting-Shou Liu, Si-Huei Lee, Chih-Hung Wang, Hao-Chang Chou, Yee Hui Yeo, Ching-Ping Tseng, Chien-Chang Lee

**Affiliations:** 1 Department of Family Medicine, Chang Gung Memorial Hospital, Taoyuan, and Chang Gung University College of Medicine, Taoyuan, Taiwan; 2 Graduate Institute of Clinical Medical Sciences, College of Medicine, Chang Gung University, Taoyuan, Taiwan; 3 Department of Medicine, National Taiwan University Hospital, Taipei, Taiwan; 4 Department of Rehabilitation and Physical Medicine, Taipei Veteran General Hospital, Taipei, Taiwan; 5 Department of Rehabilitation and Physical Medicine, National Yang-Ming University, Taipei, Taiwan; 6 Department of Emergency Medicine, National Taiwan University Hospital, Yunlin Branch, Douliou, Taiwan; 7 School of Medicine, National Defense Medical Center, Taipei, Taiwan; 8 Department of Medical Biotechnology and Laboratory Science, Chang Gung University, Tao-Yuan, Taiwan; 9 Molecular Medicine Research Center, Chang Gung University, Tao-Yuan, Taiwan; 10 Department of Epidemiology, Harvard School of Public Health, Boston, Massachusetts, United States of America; Rutgers University, United States of America

## Abstract

**Background:**

Blood culture is viewed as the golden standard for the diagnosis of sepsis but suffers from low sensitivity and long turnaround time. LightCycler SeptiFast (LC-SF) is a real-time multiplex polymerase chain reaction test able to detect 25 common pathogens responsible for bloodstream infections within hours. We aim to assess the accuracy of LC-SF by systematically reviewing the published studies.

**Method:**

Related literature on Medline, Embase, and Cochrane databases was searched up to October 2012 for studies utilizing LC-SF to diagnose suspected sepsis and that provided sufficient data to construct two-by-two tables.

**Results:**

A total of 34 studies enrolling 6012 patients of suspected sepsis were included. The overall sensitivity and specificity for LC-SF to detect bacteremia or fungemia was 0·75 (95% CI: 0·65–0·83) and 0·92 (95%CI:0·90–0·95), respectively. LC-SF had a high positive likelihood ratio (10·10) and a moderate negative likelihood ratio (0·27). Specifically, LC-SF had a sensitivity of 0·80 (95%CI: 0·70–0·88) and a specificity of 0·95(95%CI: 0·93–0·97) for the bacteremia outcome, and a sensitivity of 0·61 (95%CI: 0·48–0·72) and a specificity of 0·99 (95%CI: 0·99–0·99) for the fungemia outcome. High heterogeneity was found in the bacteremia outcome subgroup but not in the fungemia outcome subgroup.

**Conclusion:**

LC-SF is of high rule-in value for early detection of septic patients. In a population with low pretest probability, LC-SF test can still provide valuable information for ruling out bacteremia or fungemia.

## Introduction

The burden of sepsis is increasing globally. A survey conducted in USA in 2000 revealed that there were more than 650 thousand of cases of sepsis annually, with an average mortality rate of 18% [1]. Another U.S. report showed that the incidence of hospitalized patients with septicemia or sepsis had increased more than two folds in the last decade [2].

Aside from early optimization of hemodynamics [3], [4], timely adequate empirical antibiotics are a cornerstone of the sepsis treatment [3], [5]. Empirical therapy is then adjusted by the blood culture results, which provide information on causative microorganisms and in vitro sensitivity of antibiotics. Although blood culture has long been viewed as the gold standard test for the diagnosis of sepsis, it suffers from low sensitivity, prolonged turnaround time (>48 hours), and liability for contamination [6]. Efforts have been made to improve timeliness and accuracy of sepsis diagnosis. Recent advances include the development of novel clinical biomarkers [7], [8], refined clinical criteria [9], intricate algorithms [10], and molecular diagnostic methods [11].

The LightCycler SeptiFast Test (Roche Diagnostics, Mannheim, Germany) is a commercial diagnostic test utilizing real-time multiplex polymerase chain reaction (PCR). The diagnostic probes for PCR target the internal transcribed sequences situated between 16S and 23S bacterial ribosomal RNA as well as between 18S and 5·6S fungal ribosomal RNA [12]–[14]. Once the DNA of the pathogen is extracted from the blood and amplified by the LightCycler machine, a positive detection is recorded if the fluorescent signal emitted by internal hybridization probes reaches the threshold. Subsequently, a melting curve analysis is proceeded to identify the species. Overall, LightCycler SeptiFast Test is designed to detect 25 common pathogens (Table 1). The analytical sensitivity reported by the manufacturer is 100 CFU/mL for Candida glabrata, Streptococcus spp., and coagulase-negative Staphylococcus spp., and 30 CFU/mL for the others. With its broad range of detection, short turnaround time, and manufacturer-reported high sensitivity and specificity, such a molecular method might be a promising alternative to blood culture.

**Table 1:** SeptiFast® panel: pathogens detected by SeptiFast®.

Gram-negative bacteria	Gram-positive bacteria	Fungal pathogens
Escherichia coli	Staphylococcus aureus	Candida albicans
Klebsiella pneumoniae	Coagulase-negative Staphylococci†	Candida tropicalis
Klebsiella oxytoca	Streptococcus pneumoniae	Candida parapsilosis
Serratia marcescens	Streptococcus spp.‡	Candida krusei
Enterobacter cloacae	Enterococcus faecium	Candida glabrata
Enterobacter aerogenes	Enterococcus faecalis	Aspergillus fumigatus
Proteus mirabilis		
Pseudomonas aeruginosa		
Acinetobacter baumannii		
Stenotrophomonas maltophilia		

† Including S. epidermidis, S. haemolyticus, S. xylosus, S. hominis, S. cohnii, S. lugdunensis, S. saprophyticus, S. saprophyticus, S. capitis, S. pasteuri, S. warneri.

‡ Including S. pyogenes, S. agalactiae, S. mitis, S. mutans, S. oralis, S. anginosus, S. bovis, S. constellatus, S. cristatus, S. vestibularis., S. gordonii, S. intermedius, S. milleri, S. salivarius, S. sanguinis, S. thermophilus, S. parasanguinis.

Since its debut, LightCycler SeptiFast has been intensively studied. Nevertheless, the results are inconsistent. Taken individually, the sensitivity and specificity are dotted in a wide range, yet potentially worthwhile accuracy and benefits of LightCycler SeptiFast. Therefore, we aim to quantitatively synthesize current literatures by critiquing literatures, extracting data, and pooling with meta-analysis statistical methods to determine the diagnostic implication and significance of this method.

## Methods

Our systemic review and meta-analysis conformed to the methods and procedures recommended by Cochrane Collaboration on the meta-analysis of the diagnostic tests and the PRISMA (Preferred Reporting Items for Systematic Reviews and Meta-Analyses) [15], [16].

### Search Strategy

We performed a comprehensive search of literatures on the MEDLINE, EMBASE, and Cochrane databases to identify studies related to clinical utilization of LightCycler SeptiFast test for patients with suspected sepsis. We combined several search keywords to be “(multiplex PCR OR multiplex polymerase chain reaction OR septifast OR sepsitest OR vyoo) AND (sepsis OR bloodstream infection OR bacteremia OR septicemia)” from inception to June 2011. No language, study type or any other filter was set. We also searched bibliographies of retrieved full-text articles and latest reviews to include more related studies. We also searched bibliographies of retrieved articles and latest review and updated our search to October 2012 before the deploying of statistical analysis.

### Study Selection

We systematically included studies using predetermined inclusion criteria, which included: a) evaluation of the LightCycler SeptiFast test on blood specimens for diagnosing sepsis; and b) comparison of the LightCycler SeptiFast test results with reference standards, and c) sufficient information to calculate sensitivity and specificity. We excluded reviews, case reports, comments, and studies using the same dataset. Two authors independently assessed all the titles and abstracts to identify studies matching the inclusion criteria. Discrepancies on inclusion and exclusion were resolved by consensus meeting where additional reviewers were enrolled.

### Data Extraction

We piloted a data extraction from a few eligible studies and developed a comprehensive standardized data extraction form for subsequent use. Extracted data included characteristics of study design, characteristics of study patients, diagnostic method, and reference standard. More than one reference standard were used in many studies. We defined those using clinical criteria to diagnose infection as clinically-documented Infection (CDI), those using microbiological data from other specimens with or without blood culture as laboratory-documented infection (LDI), and those using blood culture alone as BC.

### Assessment of study quality

We assessed the quality of studies using the Quality Assessment of Diagnostic Accuracy Studies (QUADAS) instrument [17].

### Data Preparation and Statistical Analysis

We used the bivariate model for diagnostic meta-analysis to obtain weighted overall estimates of the sensitivity and specificity [18]. The bivariate approach models the logit-transformed sensitivity and specificity and adjusts for the negative correlation between the sensitivity and specificity of the index test that may arise from different thresholds used in different studies. A hierarchical summary receiver operating characteristic (HSROC) curve was constructed as a way to summarize the true- and false-positive rates from different diagnostic studies [19]. The area under the HSROC curve measures the overall accuracy of diagnostic tests. We also performed diagnostic odds ratio (DOR) meta-analysis. The DOR combines both positive and negative likelihood ratios and is a global measure of test performance. We quantify the extent of between-study heterogeneity by calculating the *I^2^* statistics [20]. To explore the source of heterogeneity, we defined potential relevant covariates a priori and tested these covariates one at a time in the meta-regression model. We used Egger's test for funnel plot asymmetry to test possible publication bias. Statistical analyses were conducted using STATA 11·0 (Stata Corp, College Station, TX). All statistical tests were two-sided, and statistical significance was defined as a P value less than 0·05.

## Results

### Identification of Studies

Our initial search yielded 248 citations (Appendix S1). After two rounds of inclusion and exclusion, a total of 34 primary studies including 6,012 patients (8,438 episodes) were eligible for analysis, of which 1,920 episodes (22·8%) were confirmed bacterial or fungal infection. Appendix S1 displays the literature selection process.

### Quality of the Included Studies

The studies varied in quality. Most of the study populations were representative of the target population. The diagnostic tests were deployed independently of the reference standards. We did not find differential verification of outcomes in the included studies. Because there was no unanimous standard to confirm clinically significant systemic infection, various definitions of reference standards were used and outcome misclassification was likely. Furthermore, few studies clearly mention the blinded interpretations between the LightCycler SeptiFast results and the clinical diagnosis; therefore, incorporation bias is likely. Results of risk of bias evaluation by QUADAS instrument were summarized in Appendix S2.

### Study Characteristics and Patient Populations

Details of the individual studies characteristics were summarized in Table 2. Most included studies prospectively enrolled patients with suspected sepsis from intensive care unit (ICU), emergency department (ED), and hematology and oncology unit. Studies by Casalta JP specifically targeted at patients with infectious endocarditis. Most of the included studies study on adult patients, except five studies included both children and adults and two included neonates or children. Eighteen of the 34 included studies reported accuracy data on bacteremia and fungemia separately. Various criteria were used as the reference standards, which can be grouped as three main broad categories. Ten (52·6%) studies used the preferred combined clinical and laboratory criteria. Seven studies (36·8%) chose to stick to the blood culture results. The remaining two (10·5%) used other laboratory specimens along with blood culture as the reference standard.

**Table 2:** Characteristics of the 34 included studies.

Study	Settings	Prevalence (Number)	Age	Inclusion Criteria	Outcome Definition	Sen., Spe.
Louie RF, 2008 [26]	ED, ICU, Others	0·19 (194)	Adults and elderly	Suspected sepsis	LDI	0·72, 0·94
Mancini N, 2008 [27]	Hematooncology	0·33 (103)	Adults	Febrile neutropenia	BC	0·97, 0·99
Vince A, 2008 [28]	ICU, Hematooncology	0·18 (38)	NA	Suspected sepsis after antimicrobial therapy	BC	0·43, 0·71
Casalta JP, 2009 [29]	Others	0·64 (67)	NA	Suspected infectious endocarditis	CDI	0·28, 0·96
Dierkes C, 2009 [30]	ICU	0·30 (100)	Adults and elderly	Suspected sepsis	LDI	0·77, 0·94
Lehmann LE, 2009 [31]	NA	0·21 (467)	Adults and elderly	Suspected sepsis	BC	0·61, 0·81
Lodes U, 2009 [32]	ICU	0·65 (258)	Adults and elderly	Suspected sepsis	CDI	0·15, 0·91
Lilienfeld-Toal MV, 2009 [33]	Hematooncology	0·25 (114)	Adults	Fever	BC	0·38, 0·86
Paolucci M, 2009 [34]	NA	0·24 (38)	Neonates	Suspected sepsis	CDI	0·89, 0·97
Varani S, 2009 [35]	Hematooncology	0·26 (129)	Adults and children	Suspected sepsis or febrile neutropenia	CDI	0·76, 0·83
Westh H, 2009 [36]	NA	0·13 (558)	NA	Suspected sepsis	BC	0·78, 0·82
Avolio M, 2010 [37]	ED	0·31 (144)	Adults and elderly	Suspected sepsis	CDI	0·91, 0·99
Bloos F, 2010 [38]	ICU	0·17 (236)	Adults and elderly	Suspected sepsis	BC	0·79, 0·74
Diamante P, 2010 [39]	ED	0·30 (234)	NA	Suspected sepsis	CDI	0·86, 0·99
Lamoth F, 2010 [40]	Others	0·25 (141)	Adults, elderly, and children	Febrile neutropenia	BC	0·26, 0·75
Lehmann LE, 2010 [41]	ICU	0·24 (453)	Adults and elderly	Suspected sepsis	CDI	0·83, 0·93
Maubon DL, 2010 [42]	Hematooncology	0·45 (115)	Adults and elderly	Suspected sepsis	CDI	0·51, 0·83
Regueiro BJ, 2010 [43]	ICU	0·25 (105)	Adults and elderly	Suspected sepsis	LDI	0·92, 0·97
Tsalik EL, 2010 [44]	ED	0·85 (310)	Adults and elderly	Suspected sepsis	CDI	0·20, 0·98
Wallet F, 2010 [45]	ICU	0·16 (99)	Adults	Fever or hypothermia	CDI	0·75, 0·99
Yanagihara K, 2010 [46]	ICU, ED, Hematooncology, Others	0·07 (395)	NA	Suspected sepsis	CDI	0·78, 0·94
Bravo D, 2011 a [47]	Hematooncology	0·32 (31)	Adults and elderly	Febrile neutropenia	BC	0·60, 0·95
Bravo D, 2011 b [47]	ICU	0·38 (53)	Adults and elderly	Fever	BC	0·55, 0·91
Josefson P, 2011 [48]	Others	0·12 (1085)	Adults, elderly, and children	Suspected sepsis	BC	0·38, 0·94
Kim B, 2011 [49]	NA	0·37 (70)	NA	Suspected catheter- related sepsis	BC	0·92, 1·00
Lucignano B, 2011 [50]	ICU, ED, Hematooncology, Others	0·10 (1673)	Children	Suspected sepsis	CDI	0·85, 0·92
Lodes U, 2011 [51]	ICU	0·40 (151)	Adults and elderly	Suspected sepsis	CDI	0·98, 0·99
Obara H, 2011 [52]	ICU, ED, Hematooncology, Others	0·15 (78)	Adults and elderly	Suspected sepsis	BC	0·92, 0·85
Grif K, 2012 [53]	ICU, Hematooncology, Others	0·25 (69)	Adults	Suspected sepsis	CDI	0·94, 0·98
Hettwer S, 2012 [54]	ED	0·45 (112)	Adults and elderly	Suspected sepsis	BC	0·70, 0·92
Mauro MV, 2012 [55]	Hematooncology, Others	0·41 (75)	Adults, elderly, and children	Immunocompromised patients suspected of sepsis	CDI	0·87, 0·95
Mencacci A, 2012 [56]	Others	0·81 (21)	Adults and elderly	Suspected endocarditis	CDI	1·00, 0·50
Pasqualini L, 2012 [57]	Others	0·13 (382)	Adults and elderly	Suspected sepsis	CDI	0·68, 0·92
Rath PM, 2012 [58]	NA	0·31 (225)	Adults and elderly	Suspected sepsis after abdominal surgery	BC	0·81, 0·77
Tschiedel E, 2012 [59]	ICU	0·12 (107)	Adults and children	Suspected sepsis	BC	0·92, 0·85

NA =  non-available. ED = Emergency Department. ICU = Intensive Care Unit. CDI = clinically documented infection. LDI = laboratory-documented infection. BC = blood culture. Sen. = sensitivity. Spe. = specificity.

### Diagnostic Accuracy of the LightCycler SeptiFast Test for composite bacteremia or fungemia outcome

The pooled sensitivity and specificity estimates for combined bacteremia and fungemia outcome were 0·75 (95% CI: 0·65–0·83) and 0·92 (0·90–0·95), respectively (Table 3). Specificity appears to be more consistent than sensitivity, since most tests turned out to be negative. The overall LR+ was 10·1 (95% CI: 6·83–15·0) and the overall LR- was 0·27 (0·19–0·39), revealing a superior rule-in value and moderate rule-out value. The area under the HSROC curve showed high discriminative capacity (0·93, 95% CI: 0·91–0·95), and the pooled DOR was 31·6 (95%CI: 18·9–52·9). Significant heterogeneity existed (*I^2^*  = 87·6%). Thus, pooled measures of the tests' diagnostic accuracy do not adequately describe the data.

**Table 3:** Summary of the subgroup analysis of the 34 included studies.

Variable	Studies (n)	Sensitivity (95% CI)	Specificity (95% CI)	Likelihood ratio+	Likelihood ratio-	AUROC (95% CI)	Diagnostic OR (95% CI)	*I^2^* (95% CI)	Publication bias (Egger's test p)
Combination of bacterial and fungal infection [26]–[59]	35	0·75(0·65–0·83)	0·92(0·90–0·95)	10·1(6·8–15·0)	0·27(0·19–0·39)	0·93(0·91–0·95)	31·6(18·9–52·9)	87·6(83·7–90·5)	0·025
**Bacterial infection**									
Bacteremia [27], [29], [30], [33], [34], [37], [43], [45]–[49], [51]–[53], [55]–[57]	19	0·80(0·70–0·88)	0·95(0·93–0·97)	15·9(10·4–24·3)	0·21(0·13–0·33)	0·96(0·94–0·98)	67·5(32·2–141·7)	79·3(68·4–86·5)	0·000
Reference standard									
CDI [29], [33], [34], [37], [45], [46], [52], [55]–[57]	10	0·82(0·68–0·90)	0·95(0·90–0·98)	17·0(8·2–35·3)	0·19(0·11–0·35)	0·96(0·94–0·97)	71·8(29·1–177)	65.1(31·5–82·2)	0·071
Pure blood culture [27], [47]–[49], [51], [53]	7	0·76(0·53–0·90)	0·94(0·90–0·97)	12·5(6·6–23·6)	0·26(0·12–0·57)	0·95(0·93–0·97)	43·7(12·5–152)	80·7(60·9–90·5)	0·009
Populations									
Adult or elderly [27], [30], [37], [43], [45], [47], [51]–[53], [56], [57]	12	0·84(0·75–0·91)	0·95(0·91–0·97)	17·2(9·4–31·4)	0·17(0·10–0·28)	0·96(0·94–0·98)	89·6(37·1–216)	67·5(40·6–82·3)	0·018
Settings									
Oncology and Hematology Unit [27], [34], [47]	3	0·83 (0·73–0·91)	0·94 (0·88–0·97)	13·6(2·1–87·4)	0·21 (0·06–0·71)	0·97 (0·96–0·98)	81·18 (5·28–1248·9)	80·7 (39·6–93·9)	0·395
ICU [30], [45], [47]	3	0·73(0·60–0·84)	0·93(0·86–0·97)	11·0 (4·8–25·1)	0·23(0·10–0·53)	0·83(0·77–0·89)	65·2(9·7–438)	71·9(4·8–91·7)	0·482
**Fungal infection**									
Fungemia [27], [29], [30], [33], [34], [37], [43], [45]–[49], [51]–[53], [55]–[57]	19	0·61(0·48–0·72)	0·99(0·99–0·99)	66·8(39·8–112)	0·40(0·29–0·54)	0·68(0·64–0·72)	125·1(62·7–250)	0·0(0·0–48·9)	0·030
Reference standard									
CDI [29], [33], [34], [37], [45], [46], [52], [55]–[57]	10	0·55(0·37–0·71)	0·99(0·98–0·99)	55·5(30·1–102)	0·46(0·31–0·67)	0·97(0·95–0·98)	92·2(35·6–238)	0·0(0·0–62·4)	0·150
Pure blood culture [27], [47]–[49], [51], [53]	7	0·65(0·42–0·82)	0·99(0·98–0·99)	76·0(28·0–206)	0·35(0·19–0·65)	0·77(0·73–0·81)	159(49·7–514)	0·0(0·0–70·8)	0·028
Populations									
Adult or elderly [27], [30], [37], [43], [45], [47], [51]–[53], [56], [57]	12	0·58(0·42–0·72)	0·99(0·98–0·99)	53·7(29·8–96·6)	0·43(0·29–0·62)	0·97(0·95–0·98)	103·3(42·8–249)	0·0(0·0–58·3)	0·232
Settings									
Oncology and Hematology Unit [27], [34], [47]	3	0·63(0·25–0·92)	0·99(0·97–0·99)	46·4(12·7–169)	0·43(0·18–0·99)	0·95 (0·56–0·99)	118·9(18·2–777)	0·0(0·0–89·6)	0·381
ICU [30], [45], [47]	3	0·71(0·49–0·87)	0·98(0·96–0·99)	36·9(11·9–115)	0·32(0·17–0·58)	0·97 (0·96–0·98)	133·1(29·7–596)	0·0(0·0–89·6)	0·197

AUROC = area under receiver operating characteristic curve. OR = odds ratio. CDI = clinically documented infection. ICU = intensive care unit.

### Diagnostic Accuracy of the LightCycler SeptiFast Test for bacteremia

When specifically targeting bacteremia, the accuracy of the LC-SF test improved with decreased heterogeneity (*I^2^*  = 79·3%). The pooled sensitivity was 0·80 (95% CI: 0·70–0·88), while pooled specificity was 0·95 (95% CI: 0·93–0·97). The LC-SF test also has a high rule-in value (LR+: 15·9; 95%CI: 10·4–24·3) and moderate rule-out value (LR-:0·21; 95% CI: 0·13–0·33) in detecting bacteremia. Results of the HSROC curves analysis (AUC: 0·96, 95%CI: 0·94–0·98) and DOR (67·5, 95% CI: 32·2–141·7) also revealed improved discrimination for the specific bacteremia outcome as compared to a composite bacteremia or fungemia outcome.

### Diagnostic Accuracy of the LightCycler SeptiFast Test for fungemia

**Figure 1:**
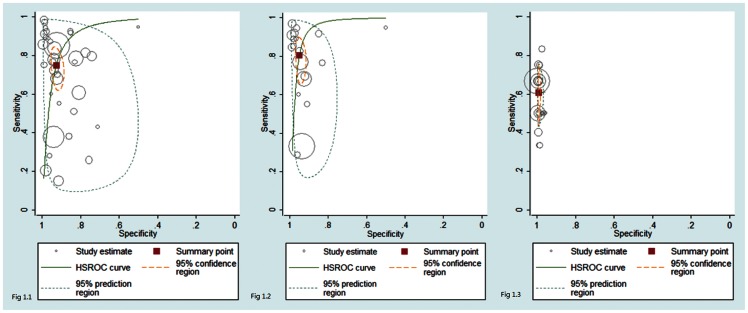
Shows the receiver operating curve analysis of the LightCycler SeptiFast molecular diagnostic method for the detection of bacterial and fungal infection (Figure 1.1), bacterial infection alone (Figure 1.2), and fungal infection alone (Figure 1.3). Solid line, solid square, inner dashed line and outer dotted line represents hierarchical summary receiver operating characteristic (HSROC) curve, bivariate summary estimate, 95% confidence ellipse, and 95% prediction ellipse. Symbol area is proportional to study size.

**Figure 2:**
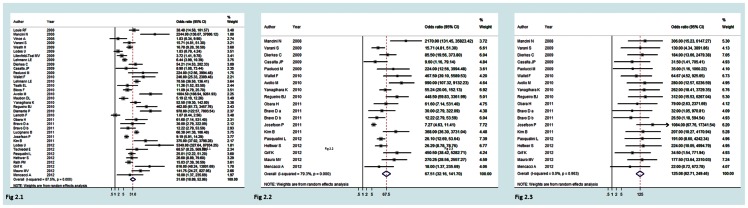
Shows forest plot of the diagnostic odds ratios of studies using the LightCycler SeptiFast diagnostic method to detect bacterial and fungal infection (Figure 2.1), bacterial infection alone (Figure 2.2), and fungal infection alone (Figure 2.3).

The performance data for the LC-SF test in detecting fungemia were available in 18 studies. Compared with the performance of the LC-SF test in detecting bacteremia, the LC-SF test had a poor sensitivity (0·61; 95% CI: 0·48–0·72) but a nearly perfect specificity (0·99; 95%: 0·99–0·99) when detecting fungemia. Results from the nineteen studies showed a similar trend with a nearly perfect heterogeneity measure (I2 = 0). The pooled LR+ was high (LR+: 66·8, 95% CI: 39·8–112), while the pooled LR- was unacceptably poor (LR-:0·40, 95% CI: 0·29–0·54). The results suggested the LC-SF test was only good for ruling in fungemia. Figure 1 shows the HSROC curves for three different outcomes and figure 2 shows the DOR from all studies for three different outcomes in forest plots.

### Subgroup Analysis

We performed subgroup analysis by restricting studies with a similar study setting and reference standard definition. For bacteremia outcome, pooled sensitivity estimates improved moderately after restriction to adult or elderly population (0·84; 95% CI, 0·75–0·91), to hematological or oncological unit patients (0·83; 95% CI, 0·73–0·91), or to studies using CDI as the reference standard (0·82; 95%CI, 0·68–0·90). Pooled sensitivity decreased appreciably after restriction to studies using blood culture (0·76; 95% CI, 0·53–0·90) as the sole reference standard. In contrast to the variable value of sensitivity in different subgroups, specificity are relatively stable in different subgroups, which suggests the high rule-in value and unreliable rule-out value of LC-SF test in detecting systemic bacterial infection. For fungemia outcome, pooled sensitivity estimates improved appreciably after restriction to ICU patients (0·71; 95% CI, 0·49–0·87) or to studies using blood culture result alone as the reference standard (0·65; 95%CI, 0·42–0·82), while decreasing appreciably after restriction to studies using CDI (0·55; 95% CI, 0·37–0·71) as the reference standard. The specificity and the LR+ are stable to different subgroup analysis, suggesting the high rule-in value of LC-SF test in detecting systemic fungal infection.

### Publication Bias and meta-regression analysis

We performed meta-regression analysis to explore source of heterogeneity and to help explain the variation after subgroup analysis (Table 4). Meta-regression analysis yielded a relative DOR for each pre-specified covariate in the model. We did not find the effect estimate significantly changed by the reference standard definition, design characteristics, study setting, and region of the study origin. There was some evidence of publication bias in the overall analysis (Egger test p = 0.025) and studies targeting bacteremia (Egger test p<0.001) or targeting fungemia (Egger test p = 0.030).

**Table 4:** Exploration of heterogeneity in assessment of accuracy of LightCycler SeptiFast test for diagnosis of bacteremia or fungemia.

Potential source of heterogeneity	Relative diagnostic odds ratio (95% confidence interval)	P-value
**Bacteremia**		
Outcome definition		
Clinically documented infection	2·60 (0·31–21·79)	0·35
Laboratory documented infection	5·10 (0·17–156·22)	0·32
Blood culture	Reference	NA
Patient group		
Adult or elderly	3·29 (0·37–28·97)	0·26
Mixed adult or pediatric patients	Reference	NA
Setting		
ICU	0·60 (0·03–10·35)	0·70
Hematologic or oncologic unit	1·63 (0·10–26·66)	0·71
Various source of patients	Reference	NA
Region		
Europe	0·31(0·02–5·26)	0·39
Other	Reference	NA
**Fungemia**		
Outcome definition		
Clinically documented infection	0·40 (0·06–2·66)	0·91
Laboratory documented infection	2·96 (0·16–55·31)	0·31
Blood culture	Reference	NA
Patient group		
Adult or elderly	0·48 (0·08–2·90)	0·89
Mixed pediatric or adult population	Reference	NA
Setting		
ICU	0·26 (0·01–4·96)	0·53
Hematologic or oncologic unit	0·76 (0·06–9·06)	0·81
Various source of patients	Reference	NA
Region		
Europe	1·09 (0·10–11·81)	0·63
Other	Reference	NA

## Discussion

Our study was designed to assess the diagnostic accuracy of the LC-SF test for detecting bacterial and fungal infection among patients suspected of infection. Our meta-analysis, which included 34 studies comprising a total of 6,012 patients, provided an overall summary of the diagnostic accuracy of the PCR methods. Overall, SeptiFast had a high specificity with a modest and highly variable sensitivity. For the clinicians, this means the rule-in value is higher than the rule-out value. In the presence of a positive SeptiFast result in a patient with suspected bacterial or fugal sepsis, a clinician can confidently diagnose bacteremia or fungemia and begin appropriate antimicrobial therapy, while forgoing unnecessary additional diagnostic testing. However, a negative SeptiFast result has a reasonable likelihood of being false-negative and should be confirmed by other clinical or laboratory diagnostic tests if the result is likely to affect patient management.

On the basis of our study, the pooled LR+ of the LC-SF test to diagnose bacterial sepsis was 15·9 (95% CI: 10·4–24·3); and the pooled LR- was 0·21 (95% CI: 0·13–0·33), which could translate into a positive post-test probability of 80% and a negative post-test probability of 5% in a virtual population with the prevalence of bacterial sepsis as 0·20 (the actual prevalence of this study was 0·19). As far as fungal sepsis was concerned, the LC-SF test had a LR+ of 66·8 (95% CI: 39·8–112), and a LR- of 0·40 (95% CI: 0·29–0·54), which could derive a positive post-test probability of 66·8% and a negative post-test probability of 1% in a virtual population with the prevalence of fungal sepsis as 0·02 (the actual prevalence of this study was 0·019). These figures help us gain further insight in their use in the clinical practice. Although the value of the LC-SF test in ruling out either systemic bacterial or fungal infection was not as good as that in ruling them in, the low background prevalence of both diseases makes these test still provide valuable rule-out information. A post-test probability as low as 5% for bacterial sepsis may justify withholding antibiotics treatment in selected cases whose LC-SF test is negative and clinical manifestation and other ancillary laboratory tests do not strongly suggest a severe infection. Likewise, although the LR- for the LC-SF to diagnose systemic fungal infection is only 0·44, the extremely low pretest probability of fungemia in most clinical setting allows the negative results of LC-SF test to remain as useful information for clinical decision. The 1% post-test probability in patients with a negative LC-SF test for fungal infection also justifies withholding anti-fungal therapy and searching for other causes of clinical deterioration and repeating the microbiological workup. If a post-test probability of negative LC-SF test of 10% is a clinically tolerable threshold for withholding antimicrobial treatment, the diagnostic value of LC-SF test would lose its reference value once the pretest probability rise to 35% for bacterial infection and 22% for fungal infection.

From the technical viewpoint, the lack of sensitivity in the LC-SF test may be attributable to insufficient concentration of bacteria and limited sets of primers in the diagnostic kit. Although it seems logical to include more primers in a diagnostic kit or to draw more blood from a patient, the blood volume allowed in a PCR machine is limited, and drawing large amount of blood from a patient may not be feasible, especially for pediatric or hematological patients. Therefore, certain modification has been suggested. Päivi T et al. [21] raised the number of bacteria or fungi in the blood by culturing the blood specimens 48 hours before deploying hybridization assay. Such a combination method was shown to effectively raise the sensitivity of a multiplex PCR-based diagnostic array to 0·95 (95% CI: 0·94–0·96) and a specificity of 0·99 (95% CI: 0·98–0·99). The cost of this strategy is the delayed turnaround time as an additional 24 to 48 hours are required for the direct LC-SF test. Another new technology that may address this problem may be the broad-range PCR amplification of conserved bacterial DNA sequences, such as the 16S ribosomal RNA (rRNA), 23S rRNA, and 16S-23S rRNA interspace regions. Numerous studies [22] have demonstrated that broad-range PCR of the conserved bacterial DNA sequences generates valuable information that complements results of time-consuming and subjective phenotypic tests for detecting bacterial infections. When real-time PCR and high-resolution melting analysis are adopted, broad-range amplification of bacterial DNA offers additional benefits including minimal labor, rapid turnaround time and a reduced risk of PCR carryover contamination.

There are three previous meta-analyses addressing the accuracy of multiplex PCR-based microbiological diagnostic methods. Carlo Mengoli et al. [23] reviewed literatures studying the diagnostic accuracy of several in-house PCR methods on patients with invasive aspergillosis and reported a pooled sensitivity of 0·88 (95% CI: 0·75–0·94) and a pooled specificity of 0·75 (95% CI: 0·63–0·84). In another study, Tomer A [24] reviewed studies targeting patients with invasive candidiasis. The pooled sensitivity was 0·95 (95% CI: 0·88–0·98), and the pooled specificity was 0·92 (95% CI: 0·88–0·95). In comparison, our results showed the commercial LC-SF test has a lower sensitivity (0·61) but higher specificity (0·99) than in-house kits when detecting fungal infection. We could not calculate the pathogen-specific accuracy data from the extracted data, but it has been shown the accuracy of PCR methods is pathogen dependent. Pammi M [25] reviewed literatures targeting pediatric patients and concluded the pooled sensitivity and specificity as 0·90 (95% CI: 0·78–0·95) and 0·96 (95% CI: 0·94–0·97), respectively. In comparison, we showed a lower sensitivity (0·75) and specificity (0·92) in our meta-analysis. We did not have a sufficient number of pediatric studies to perform subgroup analysis, but excluding several studies with mixed pediatric and adult population showed raised sensitivity in detecting bacteremia. Unless there is a head-to-head parallel comparative study, we cannot conclude whether the accuracy of PCR-based microbiological diagnosis varies among age groups.

Our study has strengths and limitations. This is the first systemic review that focuses on the accuracy of commercial real-time-PCR-based system LC-SF. Previous meta-analysis included studies using various kinds of in-house multiplex PCR kits and the results could not be readily generalized to current practice. Another major strength of our study is that we extracted, analyzed, and reported the accuracy of data on bacterial and fungal infection separately. It turned out the accuracy profile of LC-SF test in bacterial and fungal sepsis detection was drastically different. There are also several limitations in our study. First, currently, there is no evidence that LC-SF improves patient-important outcomes. Second, the higher false-negative rate of the LC-SF test still carries a potential adverse impact on patient safety. It is therefore recommended that these tests should be interpreted in the context of pre-test probability. Third, by pooling studies dealing with a variety of sample types, clinical settings, and study populations, we may have introduced heterogeneity. No major controllable factor was found to explain the heterogeneity. Lastly, at present, there is no formal cost-effectiveness analysis for the LC-SF test. If the use of LC-SF can lead to reduction of use of broad spectrum antibiotics at the early course of sepsis treatment, the additional cost may prove worthwhile.

## Conclusion

Based on the published studies, we conclude that the LC-SF test has higher rule-in than rule-out diagnostic value. In populations in which the prevalence of systemic bacterial or fungal infection is low, the negative LC-SF test still offer useful information for clinical decision. The major limitation of the LC-SF test is its suboptimal sensitivity. Before newer technology is available, we recommend clinicians combine biomarkers, clinical findings, and the LC-SF test to enhance the diagnostic accuracy.

## Supporting Information

Appendix S1 **The flow chart shows the procedure used by the current systematic review to identify studies using the LightCycler SeptiFast molecular diagnostic method to detect bacterial or fungal infection.**
(TIF)

Appendix S2 **The figure shows QUADAS (Quality Assessment of Diagnostic Accuracy Studies) criteria for the included studies.**
(TIF)

Checklist S1 **PRISMA Checklist.**
(DOC)
