# Supplementary figures and images for: Multiplex PCR System for Rapid Detection of Pathogens in Patients with Presumed Sepsis – A Systemic Review and Meta-Analysis
Source: PLoS One. 2013 May 29;8(5):e62323. doi: 10.1371/journal.pone.0062323 (PMC3667030; doi:10.1371/journal.pone.0062323)

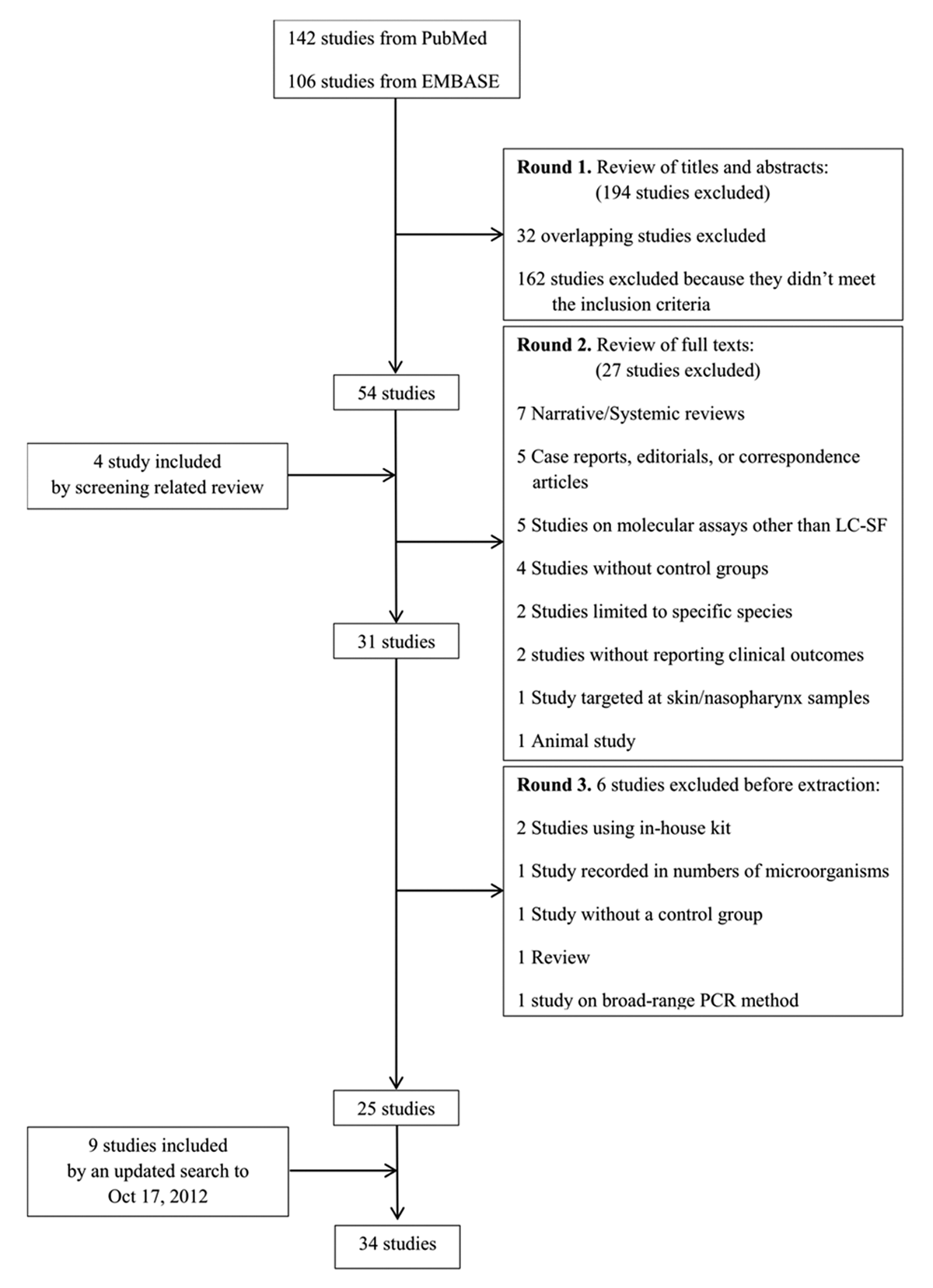

Supplement: Appendix S1 — The flow chart shows the procedure used by the current systematic review to identify studies using the LightCycler SeptiFast molecular diagnostic method to detect bacterial or fungal infection. (TIF) [file pone.0062323.s001.tif]

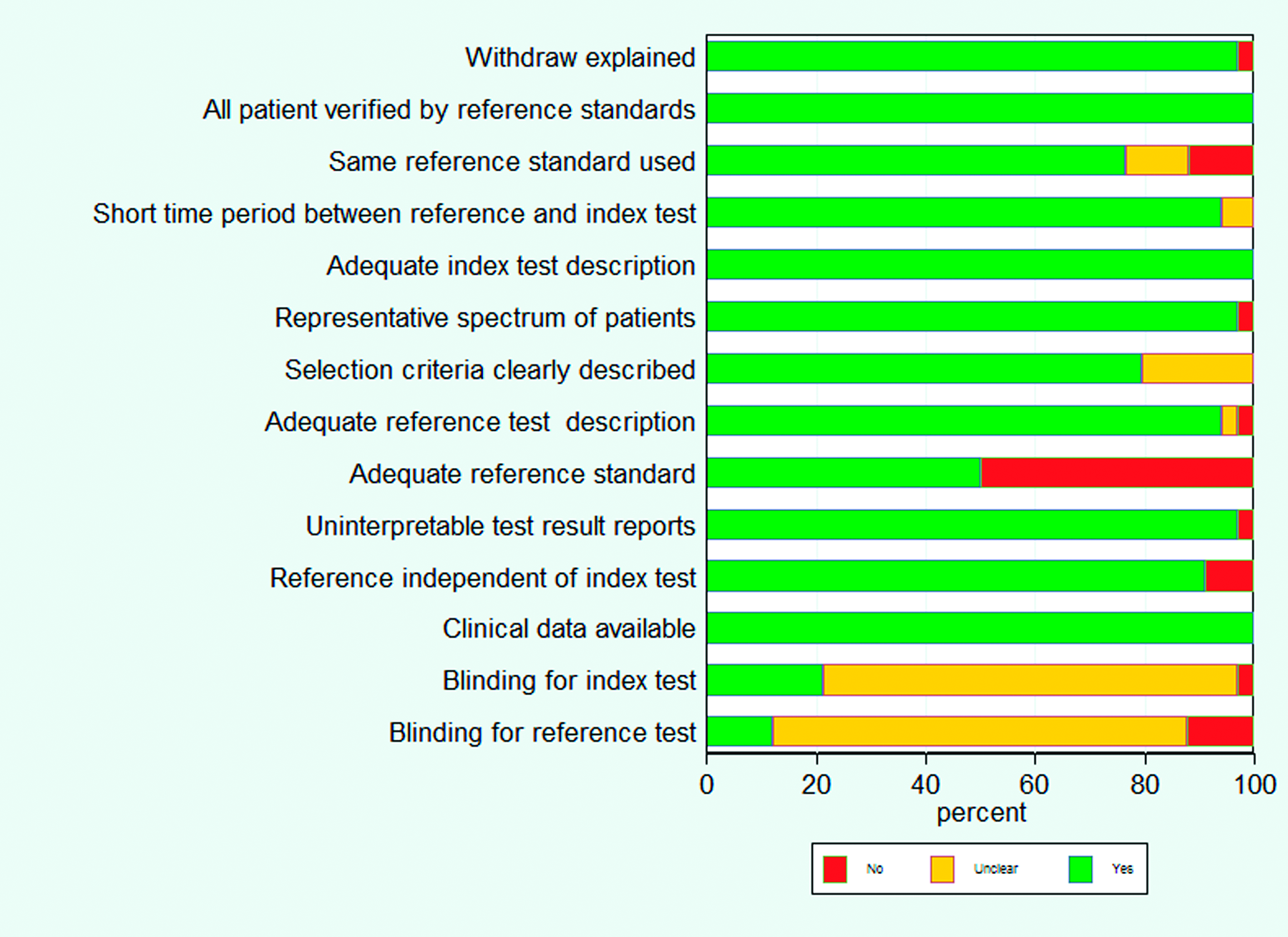

Supplement: Appendix S2 — The figure shows QUADAS (Quality Assessment of Diagnostic Accuracy Studies) criteria for the included studies. (TIF) [file pone.0062323.s002.tif]
